# Treatment adherence and persistence with long-acting somatostatin analog therapy for the treatment of acromegaly: a retrospective analysis

**DOI:** 10.1186/s40360-017-0124-y

**Published:** 2017-04-04

**Authors:** Michelle H. Gurel, Yi Han, Andrea L. Stevens, Aaron Furtado, David Cox

**Affiliations:** 1 Neuroendocrine and Pituitary Tumor Clinical Center, Massachusetts General Hospital, Zero Emerson Place, Suite 112, Boston, MA 02114 USA; 2 WG Consulting, 200 Fifth Ave, New York, NY 10010 USA; 3 Ipsen Biopharmaceuticals, Inc, 106 Allen Road, Basking Ridge, NJ 07920 USA

**Keywords:** Acromegaly, Lanreotide depot, Long-acting, Octreotide, Somatostatin analog, Treatment adherence, Treatment persistence

## Abstract

**Background:**

Many patients with acromegaly require medical treatment that includes somatostatin analogs (SSAs). Long-acting SSA formulations are widely used, due in part to increased patient convenience and increased treatment adherence vs daily medications. Although medication compliance can be poor in patients with chronic conditions, adherence and persistence with these SSAs in patients with acromegaly has not been evaluated. This analysis utilized claims data to estimate treatment adherence and persistence for lanreotide depot and long-acting octreotide in this population.

**Methods:**

This retrospective analysis used the MarketScan® database (~100 payors, 500 million claims in the US), which was searched between January 2007 and June 2012 to identify patients with acromegaly taking either lanreotide depot or long-acting octreotide. Patients switching treatments were excluded. Treatment adherence was assessed using medication possession ratio (MPR; number of doses dispensed in relation to dispensing period; ≥80% is considered adherent), injection count, and treatment time. Persistence was estimated by Kaplan-Meier analyses and Cox proportional hazards modeling. A washout period, defined as no acromegaly-related prescription activity 180 days prior to the index date, was employed to minimize effects of prior therapy and focus on patients more likely to be treatment-naïve.

**Results:**

Altogether 1308 patients with acromegaly receiving a single SSA for treatment (1127 octreotide, 181 lanreotide) who had not switched treatments were identified. Mean MPR in patients with a 180-day washout (*n* = 663) was 89% for those receiving octreotide (*n* = 545) and 87% for those receiving lanreotide (*n* = 118). Median number of days on therapy was 169 (95% CI 135–232) for octreotide patients and 400 (95% CI 232–532) for lanreotide patients. The point estimate of the Cox proportional hazard ratio for stopping treatment was 1.385 for octreotide vs lanreotide (95% CI 1.079–1.777), suggesting a 38.5% increased risk for stopping octreotide before lanreotide.

**Conclusions:**

Treatment adherence was similarly good for both injectable SSA treatments studied, at 87% or greater. Persistence was greater with lanreotide than octreotide and the risk of discontinuing therapy was lower with lanreotide than octreotide. Further studies to determine factors leading to these differences in persistence or to predict discontinuation of therapy may aid in clinical management of these patients.

## Background

Acromegaly is a disorder caused by hypersecretion of growth hormone (GH). It has an incidence of approximately 3 per million per year, a prevalence of 40–1000 per million, a mean age at diagnosis of 44 years, and an equal distribution between men and women [1–3]. Common clinical features of acromegaly include acral enlargement, maxillofacial changes, excessive sweating, arthralgias, headache, hypogonadal symptoms, visual defects, fatigue, and weight gain [4]. The most frequent cause of acromegaly is a GH-secreting pituitary adenoma, which can be evaluated via magnetic resonance imaging (MRI) once clinical signs and biochemical evidence of GH hypersecretion are determined [5]. The primary therapy for most patients is surgery, which will immediately lower the level of GH [1, 5, 6]. The remission rate for resected microadenomas and macroadenomas is >85 and 40–50%, respectively, with a 5-year recurrence rate of 2–8% [6].

For patients who are poor candidates for surgery, who have unresectable tumors, or who have incomplete resection of their tumor and persistent disease, the Endocrine Society recommends therapy with either a somatostatin analog (SSA) or pegvisomant, a GH receptor antagonist; for patients with mild symptoms and modest elevations in insulin-like growth factor (IGF-1), which is the main GH-dependent growth factor increased in patients with acromegaly, the recommendations is a dopamine agonist [1, 6]. The American Association of Clinical Endocrinologists recommends therapy with SSAs following surgery in patients with residual disease, dopamine agonists in patients with mild biochemical activity, and pegvisomant in patients with incomplete responses to SSA or dopamine agonists [5]. Lanreotide and octreotide are long-acting SSAs given as injections every 4 weeks as long-term therapy for patients with an inadequate response to surgery or who are not candidates for surgery [7–9]. The long-acting formulations circumvent the daily injections required with other agents used in the treatment of acromegaly, such as pegvisomant [6]. There is no clear benefit on surgical outcomes or perioperative morbidity/mortality with use of SSAs prior to surgery [10].

Effective therapy requires adherence, defined as taking a medication at the time it is prescribed, and persistence, defined as the duration between starting and discontinuing a medication [11, 12]. For retrospective analyses, adherence is defined as the number of doses dispensed in relation to the dispensing period, or the medication possession ratio (MPR) [12]. Unfortunately, adherence and persistence are often difficult to maintain in patients with chronic conditions, with an estimated nonadherence rate of 25–50% across multiple chronic conditions [13]. The adherence and persistence for lanreotide and long-acting octreotide in patients with acromegaly has not been established, and therefore the primary objective of this analysis was to determine the adherence and persistence of SSAs in patients with acromegaly using a retrospective database of pharmacy claims to reflect real-world clinical practice. For this analysis, adherence was measured by MPR and persistence was estimated by Kaplan-Meier and Cox proportional hazards modelling. This analysis also investigated the effect of a treatment washout period on both adherence and persistence.

## Methods

### Study design

This retrospective analysis, conducted in 2013, used the Truven Health Analytics MarketScan® database, which links paid claims and encounter data for more than 100 payers and 500 million claims the United States. The database includes commercial claims and encounters (CCAE) from active employees, early retirees, COBRA continuees, and dependents insured by employer-sponsored plans. The number of patients captured in the Truven database was not known. The database was reviewed for the period between January 2007 and June 2012 to identify medical encounters due to acromegaly and patients with acromegaly receiving either lanreotide depot (Somatuline® depot, Ipsen Biopharmaceuticals, Inc., Basking Ridge, NJ, USA) or octreotide long-acting release (Sandostatin® LAR, Novartis Pharmaceuticals, East Hanover, NJ, USA). All dosages were included in the analysis. Patients were assumed to be receiving the proper dosage and expected to continue with therapy. Medical claims with an ICD-9 diagnosis code for acromegaly or a pharmacy claims for lanreotide, octreotide, or pegvisomant were included in the initial scan. The ICD-9 code included was 253.0. Because the analysis is a by-product of a market share analysis, pegvisomant was initially included in the persistence analysis, but was removed later in the analysis because it belongs to a different drug class. All claims within the database were unique with no duplicates. Each claim could be separated into multiple line level entries. Patients known to have switched SSA treatments within the time of the analysis were excluded. Data unavailable for SSA injections given prior to the start of the data collection period, and thus some patients may have been treated previously with another therapy. To exclude the effect of any SSA injections that may have been administered prior to the start of the data collection period, a 180-day washout period was used. During the washout period, patients were not allowed to take acromegaly-related prescriptions, but were permitted to take other medications. The washout period was defined as the number of days between the initial occurrence of the patient in the database and the first injection of an SSA. Use of the washout period focused on data from patients more likely to be treatment-naive patients.

### Statistical analyses

Treatment adherence was assessed for each patient by determining the MPR. Patients were defined as adherent if the amount of medication provided to the patient covered at least 80% based on days’ supply of medication divided by the number of days the patient should be consuming the medication, using the equation ${MPR}_i=\left({NI}_i-1\right)*30/\left({TL}_i- T{1}_i\right)$ where *NI* was the total number of injections for patient *I*, and where *T*1 and *TL* were the times for the first injection and last injection, respectively. A factor of 30 was introduced as the sum of 28 days of treatment per injection with a 2-day grace period between refills.

Persistence to treatment was analyzed using survival analysis, a time series statistical method used to model time-to-event data [14]. For this study, a persistent event was defined as the first occurrence of a patient more than 15 days overdue for injection. The persistence was calculated as $S(t)= \Pr \left( T> t\right)={\int}_t^{\infty } f(u) d u=1- F(t)$ where *S(t)* was the survival function, Pr was probability, T was a random variable denoting the time of the event, *t* was a period of time, ƒ(*u*) was the density function and *F*(*t*) was the cumulative distribution function. The hazard function or probability of an event occurrence, λ(*t*), was calculated as $\lambda (t)=\underset{dt\to 0}{ \lim}\frac{ \Pr \left( t\le T< t+\left. dt\right| T\ge t\right)}{dt}=\frac{f(t)}{S(t)}=\frac{- S^{\prime }(t)}{S(t)}$ where Pr was the probability of event occurrence and ƒ(*t*) was the lifetime density function.

This study applied a non-parametric Kaplan-Meier model and a semi-parametric Cox proportional hazards model. For the Kaplan-Meier model, the patient was considered to have an event if not treated within 30 days of the last treatment. Patients were right-censored if the database stopped capturing the patient’s data <30 days from the last injection. Whether patients were treated with an extended schedule of lanreotide, as Food and Drug Administration (FDA)-approved, was not considered for the analysis. The criteria for measuring persistence was applied regardless of whether a patient was on an extended dosing interval. The extended dosing interval is FDA-approved only for lanreotide, so a comparative analysis for the proportion of adopted extended schedules between octreotide and lanreotide patients could not be performed. The study did not capture the relation of SSA treatment adherence with radiation therapy. The Kaplan-Meier method could fully utilize information provided by right-censored data. Median survival time was the point at which 50% of the patients were still adherent to treatment. Median survival time could be directly read from the Kaplan-Meier curve.

The proportional hazards model attributed the survival time to one or more contributing factors, with the actual hazard calculated from a baseline hazard model λ_0_ and exponent from risk factors with treatment with octreotide or lanreotide as the risk factors with the equation: $\lambda \left(\left. t\right| X\right)={\lambda}_0(t) \exp \left({\beta}_{1\;}{X}_1+\cdots +{\beta}_p{X}_p\right)={\lambda}_0(t) \exp \left({\beta}^{\prime } X\right)$. The relative risk of stopping treatment or hazard ratio (HR) was estimated from the model, with a HR >1 indicating an increased risk of stopping treatment, a HR =1 indicating an equal risk of stopping treatment, and a HR <1 indicating a reduced risk of stopping treatment.

## Results

### Patients

**Fig. 1:**
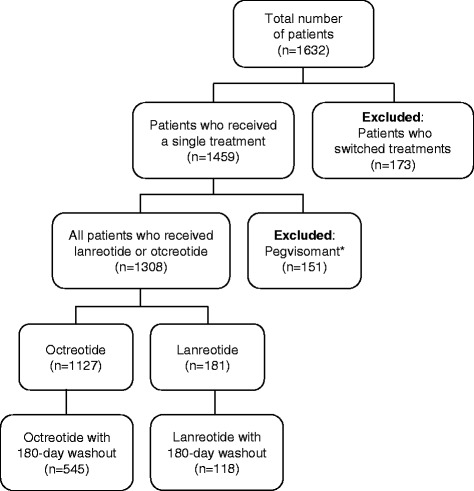
Patient selection. *Pegvisomant was excluded from the analysis because it is not a somatostatin analog and the mode of treatment (daily injection) was not considered comparable to that of octreotide and lanreotide (monthly injection) for this study

**Fig. 2:**
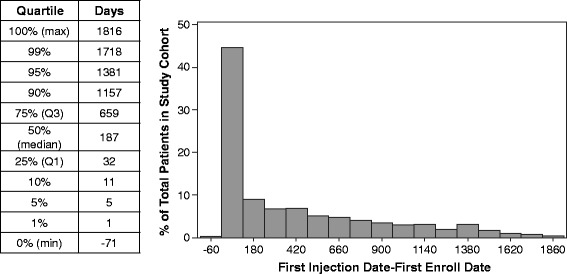
Distribution of patients identified in the database throughout the study period

The search of the MarketScan database between January 2007 and June 2012 identified 1632 unique patients treated for acromegaly with octreotide, lanreotide, or pegvisomant, (Fig. 1). Of these patients 1459 received a single type of therapy, and 1308 who had not switched medications received a long-acting SSA (1127 octreotide, 181 lanreotide). There were 17,827 prescriptions for octreotide, pegvisomant, or lanreotide, with 3395 of these prescriptions for outpatient injections of octreotide, lanreotide, or pegvisomant associated with a diagnosis code for acromegaly. No inpatient treatment with these medications for acromegaly was identified. The number of patients identified in the longitudinal claims database was not evenly distributed over the total period of follow-up (Fig. 2). Limited demographic information was available from the database search, but the age and gender were similar for both treatment cohorts and independent of the inclusion of the washout period (Table 1).

**Table 1:** Age and gender for patients with acromegaly receiving octreotide or lanreotide

		All patients			Patients with a 180-day washout period		
Therapy	Variable	N	Mean	SD	N	Mean	SD
Octreotide	Age at first injection (yr)	1127	50.6	11.1	545	51.4	11.2
	% of male patients	1127	45.9%	0.5	545	48.1%	0.5
Lanreotide	Age at first injection (yr)	181	45.9	11.9	118	45.7	11.7
	% of male patients	181	45.9%	0.5	118	44.9%	0.5

*N* sample size, *SD* standard deviation

### Adherence to lanreotide and octreotide in patients with acromegaly

Descriptive statistics for the number of injections, treatment time, and MPR were calculated for each treatment group (Tables 2 and 3). The MPR for all patients receiving octreotide or lanreotide (*n* = 1308) was 85 and 87%, respectively, for those receiving octreotide and lanreotide. The MPR for patients with a 180-day washout period (*n* = 663) was 89 and 87%, respectively, for those receiving octreotide and lanreotide, suggesting no effect of the washout period on adherence to therapy with an SSA. In the analysis of adherence outcomes in all patients (Table 2), there were no differences in the number of injections (10.8 octreotide vs 11.1 lanreotide) or mean time of treatment (418.5 vs 383.8). In the analysis of patients with a washout period (Table 3), there were fewer injections of octreotide (7.21 vs 10.2) and a shorter treatment time in patients receiving octreotide (254.7 days vs 351.8 days).

**Table 2:** Treatment adherence outcomes in all patients with acromegaly receiving octreotide or lanreotide

Treatment	N	Variable	N	Mean	SE	Minimum	Maximum
Octreotide	1127	Treatment time (days)	1127	418.5	14.9	0	1819
		Number of injections	1127	10.8	0.4	1	68
		MPR (%)	891	87	4	0.06	30
Lanreotide	181	Treatment time (days)	181	383.8	28.1	0	1454
		Number of injections	181	11.1	0.8	1	49
		MPR (%)	155	85	3	0.12	3.8

Only one injection was logged and used for treatment time calculation

*N* sample size, *SE* standard error, *MPR* medication possession ratio

**Table 3:** Treatment adherence outcomes in patients with acromegaly receiving octreotide or lanreotide with 180-day washout period

Treatment	N	Variable	N	Mean	SE	Minimum	Maximum
Octreotide	545	Treatment time (days)	545	254.7	14.5	0	1563
		Number of injections	545	7.21	0.4	1	63
		MPR (%)	389	89	3	0.06	10
Lanreotide	118	Treatment time (days)	118	351.8	34.2	0	1454
		Number of injections	118	10.2	0.9	1	49
		MPR (%)	99	87	4	0.13	3.8

*N* sample size, *SE* standard error, *MPR* medication possession ratio

### Persistence with lanreotide and octreotide in patients with acromegaly

**Fig. 3:**
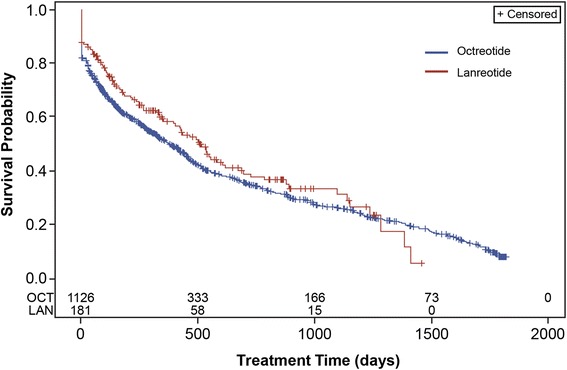
Kaplan Meier analysis of treatment persistence with octreotide vs lanreotide, all patients (*n* = 1308). Numbers above axis indicate subjects at risk. Survival indicates probability of patients continuing treatment. *LAN* lanreotide; *OCT* octreotide

**Fig. 4:**
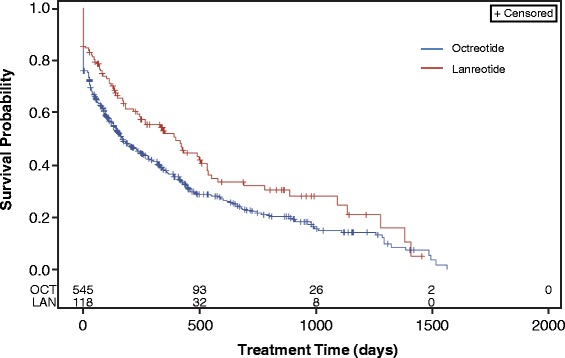
Kaplan-Meier analysis of treatment persistence with octreotide or lanreotide, patients with 180-day washout period (*n* = 663). Numbers above axis indicate subjects at risk. Survival indicates probability of patients continuing treatment. *LAN* lanreotide; *OCT* octreotide

**Fig. 5:**
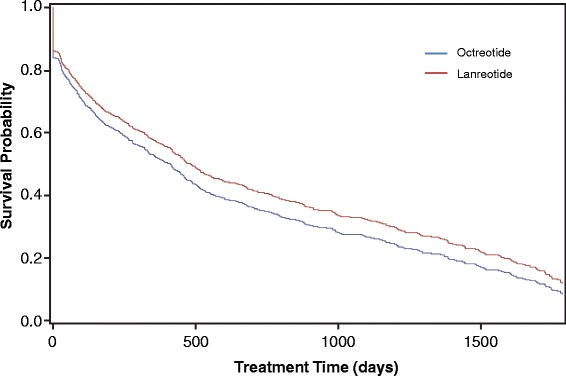
Persistency chart (proportional hazards model) for octreotide vs lanreotide, all patients (*n* = 1308). Survival indicates probability of patients continuing treatment. Hazard ratio (risk of discontinuing treatment) for octreotide vs lanreotide: 1.164; *p* = 0.155

**Fig. 6:**
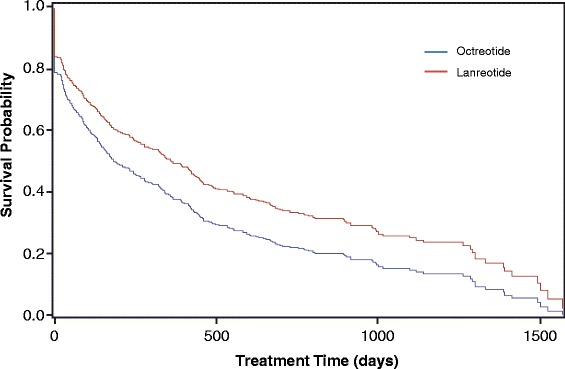
Persistency chart (proportional hazards model) for octreotide vs lanreotide, patients with 180-day washout period (*n* = 663). Survival indicates probability of patients continuing treatment. Hazard ratio (risk of discontinuing treatment) for octreotide vs lanreotide: 1.381; *p* = 0.011

The mean persistence, measured by Kaplan-Meier analysis, was 379 days (95% CI 325, 430) for all patients receiving octreotide and 506 days (95% CI 392, 617) for all patients receiving lanreotide (Fig. 3, *n* = 1308). When measured by Kaplan-Meier analysis with the 180-day washout, the mean persistence was 169 days (95% CI 135, 232) for patients receiving octreotide and 400 days (95% CI 232, 532) in patients receiving lanreotide (Fig. 4, *n* = 663). In all patients, a Cox proportional hazards model indicated no difference in the likelihood of discontinuing either lanreotide or octreotide (HR: 1.164; *p* = 0.155) (Fig. 5). In patients with a washout period, a Cox proportional hazard model showed a significant increase in risk of discontinuing octreotide compared with lanreotide (HR: 1.381; *p* = 0.011) (Fig. 6).

## Discussion

We found that the risk of discontinuing octreotide was greater than lanreotide in this analysis of “real world” acromegaly data. The potential implications are important, since adherence and persistence are necessary for these drugs to work effectively. The use of the MarketScan database enables a longer-term assessment of adherence in a less structured setting vs clinical trials. Patients enrolled in the three trials that led to the approval of octreotide were treated for either 12 or 28 months (52 or 112 weeks) and patients enrolled in the clinical trials with lanreotide were treated for either 48 or 52 weeks [8, 9]. In this retrospective analysis, the median persistence for treatment was 169 days (5.6 months) with long-acting octreotide and 400 days (57.1 weeks) with lanreotide depot. Why treatment persistence was different between the two drugs in this setting is worth further exploration.

This analysis utilized the MarketScan database to retrospectively study the adherence and persistence of treatment with either lanreotide or octreotide in patients with acromegaly. While the initial data search included patients being treated with pegvisomant, a daily GH agonist, there were ultimately only 1308 patients identified who had been treated with a single SSA, with only 181 of these patients taking lanreotide over the 5.5-year data collection period before the analysis was limited with a washout period.

This analysis excluded patients who had received more than one therapy for treatment of acromegaly. It may be worth considering including these patients in future studies, particularly since this may help explain differences in adherence and persistence, such as reasons for discontinuing an SSA or starting a different one. The current analysis establishes a comparison of adherence and persistence in a set of patients who are likely receiving first-line therapy with an SSA due to the exclusion of more than one therapy and inclusion of a washout period. Using these methods for analysis of adherence and persistence with a more inclusive dataset would also be more reflective of actual clinical practice and increase the number of patients in the analysis.

The washout period was included in this analysis in order to minimize the likelihood of an effect of any SSA injections prior to the start of the data collection period. There is no standard length for a washout period with previous reimbursement claim analysis studies, which have used a 6-month or 12-month washout period [15, 16]. A longer washout period more effectively isolates treatment effects; however, this is at the cost of reducing the sample size. For this analysis, a washout period of 180 days was selected to improve data quality, which significantly decreased the sample size.

An interesting finding from this analysis was that the inclusion of a washout period altered the persistence or likelihood to discontinue treatment with a SSA in the absence of an effect on adherence. This effect was not on the adherence, the parameter more temporally related to the start of therapy and the potential influences of previous therapies. The effect of the washout was on how long patients remained on therapy. In this analysis, there was no difference in adherence, measured by MPR, between patients receiving lanreotide and octreotide regardless of the inclusion of a washout period. In contrast, the persistence of therapy with lanreotide and a decrease in the likelihood of discontinuing lanreotide was only observed when a 180-day washout was incorporated. Possible differences between lanreotide and octreotide that may impact adherence and long-term persistence include a subcutaneous route of administration for lanreotide, an intramuscular injection for octreotide, and the availability of lanreotide in a prefilled syringe compared with the need to dilute octreotide prior to injection [8, 9]. While these factors may confer a clinical advantage favoring lanreotide in some patients, it is not clear from the retrospective data analysis reported here that these factors would be important for differences in persistence of SSA therapy due to inclusion of a washout period. The reason for this washout-associated difference in persistence should be explored. Further, addition of clinical information about reasons for discontinuation of therapy, any further therapy the patients who discontinue therapy with an SSA may receive, and persistence to the second therapy may be useful for clinicians as they chose an initial and second therapy for their patients.

When determining the clinical impact of this analysis, the primary limitations include the number of patients included in the analysis, the exclusion of patients who had switched therapy, and the lack of clinical data from these patients. Acromegaly is a rare disease, and the analysis of the database over a 5.5 year period only captured 1,308 patients who had received a single SSA. The number of covered lives in the database was not available, but the number is less than what would be expected based on the incidence and prevalence of the disease. Possible explanations for the lower number of prescriptions on average may be that patients changed medical plans or medical plans stopped reporting data to Truven. The use of the 180-day washout period and the exclusion of patients who switched therapies contributed to the low accrual. The combination of adherence and persistence data with clinical information may lead to interesting findings impacting the use of each SSA if drug toxicity or a lack of response lead to decreased adherence and persistence due to a need to change therapy. Finally, inclusion of clinical data, particularly at the time that drug was discontinued, could provide very important information to understand the finding of this analysis. In these patients, the adherence was not significantly different for the two drugs and this likely reflects similar tolerance. In the absence of clinical data, it is unclear if the increased persistence is good or bad. A shorter persistence on therapy with octreotide due to toxicity would be favorable for use of lanreotide, while a longer persistence on lanreotide due to less effectiveness to control the acromegaly would support use of octreotide. The incorporation of clinical data would answer these questions. Otherwise, the persistence is longer in patients receiving lanreotide in the setting of a washout period, but while the clinical impact can be inferred, it isn’t clear.

This analysis was a by-product of a market share analysis so the octreotide and lanreotide patients could not be directly compared. Without the addition of a matching step using propensity scores, it could only be assumed that the octreotide and lanreotide treatment groups were comparable. Because we identified patients by diagnosis codes and treatments, there was no indication of whether the patients were on extended schedules or in withdrawal in the claim database. There was no evidence the extended schedule or withdrawal period were happening disproportionally between two treatment groups. Therefore, we did not stratify on that variable, which may be a potential confounder in the interpretation of the results.

## Conclusions

This study has shown that a retrospective database can be used to determine adherence to therapy, measured by MPR, and to compare the persistence to treatment by modelling with Kaplan-Meier and Cox proportional hazards modelling in patients receiving SSAs for acromegaly. In this analysis, patients receiving lanreotide and octreotide had similar adherence to therapy, with an increase in persistence for patients receiving lanreotide compared with octreotide when a washout period was included. Further studies are warranted to determine what factors lead to increased time on therapy to guide clinicians treating this disease.

## Abbreviations

CCAE: Commercial claims and encounters
CI: Confidence interval
COBRA: Consolidated omnibus budget reconciliation act
FDA: Food and drug administration
GH: Growth hormone
HR: Hazard ratio
IGF-1: Insulin-like growth factor 1
LAN: Lanreotide
MPR: Medication possession ratio
MRI: Magnetic resonance imaging
OCT: Octreotide
SD: Standard deviation
SSA: Somatostatin analog
